# Impact of multidisciplinary team on the pattern of care for brain metastasis from breast cancer

**DOI:** 10.3389/fonc.2023.1160802

**Published:** 2023-08-16

**Authors:** Fei Xu, Dan Ou, Weixiang Qi, Shubei Wang, Yiming Han, Gang Cai, Lu Cao, Cheng Xu, Jia-Yi Chen

**Affiliations:** Department of Radiation Oncology, Ruijin Hospital, Shanghai Jiao Tong University, School of Medicine, Shanghai, China

**Keywords:** brain metastases, breast cancer, MDT, pattern of care, intracranial RT, systemic therapy

## Abstract

**Purpose:**

The aim of this study was to explore how a multidisciplinary team (MDT) affects patterns of local or systematic treatment.

**Methods:**

We retrospectively reviewed the data of consecutive patients in the breast cancer with brain metastases (BCBM) database at our institution from January 2011 to April 2021. The patients were divided into an MDT group and a non-MDT group.

**Results:**

A total of 208 patients were analyzed, including 104 each in the MDT and non-MDT groups. After MDT, 56 patients (53.8%) were found to have intracranial “diagnosis upgrade”. In the matched population, patients in the MDT group recorded a higher proportion of meningeal metastases (14.4% vs. 4.8%, *p* = 0.02), symptomatic tumor progression (11.5% vs. 5.8%, *p* = 0.04), and an increased number of occurrences of brain metastases (BM) progression (*p <* 0.05). Attending MDT was an independent factor associated with ≥2 courses of intracranial radiotherapy (RT) [odds ratio (OR) 5.4, 95% confidence interval (CI): 2.7–10.9, *p* < 0.001], novel RT technique use (7.0, 95% CI 3.5–14.0, *p <* 0.001), and prospective clinical research (OR 5.7, 95% CI 2.4–13.4, *p <* 0.001).

**Conclusion:**

Patients with complex conditions are often referred for MDT discussions. An MDT may improve the qualities of intracranial RT and systemic therapy, resulting in benefits of overall survival for BC patients after BM. This encourages the idea that treatment recommendations for patients with BMBC should be discussed within an MDT.

## Background

Breast cancer (BC) is the prevalent malignancy among women, accounting for 30% of new cancer cases (1). It is also the leading cause of cancer-related deaths in women, contributing to 15% of mortality from female cancers (2). Previous research revealed that approximately 10%–30% of patients with BC ultimately develop brain metastasis (BM) (3), which was associated with a poor prognosis (4).

The multidisciplinary team (MDT) strategy has been a standard pattern of care for patients with newly diagnosed malignant tumors. Previous work in esophageal cancer confirmed that MDTs increased twice in chances of multimodality treatment and resulted in a better overall survival (OS) (5). In an international survey of 39 countries, MDTs were considered to improve the overall quality of treatment and produce more evidence-based treatment decisions in BC (6). In the United Kingdom, MDTs helped newly diagnosed BC by monitoring non-implementation reasons to make optimal patient-centered decisions (7). In advanced BC patients with BM, the therapeutic strategy includes local treatment [surgery/radiotherapy (RT)] and systemic treatment (chemotherapy/targeted agents/endocrine therapy) (8). In a large-sample study, multidisciplinary treatment was an independent factor for decreased BM death risk in patients (9).

Systemic therapy based on molecular subtypes as well as local therapy, including different choices of radiotherapeutic and surgical techniques, has greatly altered the landscape of therapeutic strategies for patients with BM from BC. Whether an efficient MDT would affect the pattern of care regarding different lines of treatment in these patients is unknown. The present study aims to analyze the influence of MDT on local and systematic therapeutic strategies for breast cancer with brain metastases (BCBM) patients.

## Methods

### Study population

We retrospectively reviewed the consecutive BC patients with BM diagnosed in Ruijin Hospital, Shanghai Jiaotong University, School of Medicine between January 2011 and April 2021. Two MDTs that were involved in the treatment decision of patients with BCBM: the MDT of Breast Disease Center within the same period, and the MDT for metastatic central nervous system tumors, which was established in June 2019. Each MDT met every 7 days, and the participating physicians had the following disciplines depending on patient history: surgical/radiation/medical/neuro-oncology, radiology/interventional radiology, pathology, pain specialties, and nutritional support when needed.

A complete record of the treatment decisions made by the MDT, including any findings of new intra- or extracranial metastatic lesions beyond the recognition of the treating physician, was given to the patients. Local treatment recommendations included brain surgery, whole-brain radiation therapy (WBRT) or WBRT with hippocampal avoidance (WBRT-HA), stereotactic radiosurgery (SRS)/fractionated stereotactic radiotherapy (fSRT), or cranio-spinal irradiation (CSI). Systemic treatment recommendations included endocrine therapy, chemotherapy/targeted therapy, participation in a clinical trial, and supportive care. New agents are defined as those indicated for metastatic BC but not yet covered by national healthcare in China before 2019, which includes pertuzumab, trastuzumab, TDM-1, pyrotinib maleate tablets, CDK4/6 inhibitors, and PD-1/PDL-1 inhibitors in the current study. Breast-GPA was used to quantify the prognosis based on age at diagnosis of BM, number of intracranial metastases, extracranial metastases, molecular type, and KPS (10). Prospective clinical trials (PCTs) refer to registration trials of new anti-cancer agents (**Supplementary Table 1**). In the MDT group, if new lesions were discovered after the MDT meetings, they were recorded as “diagnosis updated”.

### Statistical analysis

Post-brain metastasis OS (post-BM OS) was defined as the interval from the date when the first BM was diagnosed to the date of death or the last follow-up.

Statistical analyses were performed using IBM SPSS for Mac, version 26.0. (SPSS, Chicago, IL). The demographic, clinicopathological, and treatment characteristics of the patients recorded as categorical variables were summarized and compared between those who entered in MDT discussion and those who did not, utilizing the chi-squared test at a significance level of 0.05 (**Tables 1**, **2**). Odds ratios (ORs) were estimated by logistic regression modeling. To estimate the odds ratios associated with various patient characteristics for receiving intracranial RT or participation in clinical trials, a multivariable logistic regression analysis was conducted (**Tables 3**–**5**). Survival curves were constructed using the Kaplan−Meier method and compared using the log-rank test. *p* < 0.05 was considered statistically significant.

**Table 1:** Baseline clinicopathological characteristics at the presentation or at the initial BM.

Characteristics	All	MDT	Non-MDT	*p-*value
	*n* = 211(%)	*n* = 105(%)	*n* = 106(%)	
At the first presentation				
Molecular subtype				0.14
**A**	23 (10.9)	10 (4.7)	13 (6.2)	
**B(HER2−)**	45 (21.3)	26 (12.3)	19 (9.0)	
**B(HER2+)**	39 (18.5)	23 (10.9)	16 (7.6)	
**HER2+**	47 (22.3)	25 (11.8)	22 (10.4)	
**TN**	57 (27.0)	21 (10.0)	36 (17.1)	
ER/PR				0.2
**Negative**	104 (49.3)	47 (22.3)	57 (27)	
**Positive**	107 (50.7)	58 (27.5)	49 (23.2)	
HER2 status				0.16
**Negative**	125 (59.2)	57 (27.0)	68 (32.2)	
**Positive**	86 (40.8)	48 (22.7)	38 (18.0)	
NAST				0.3
**No**	173 (82.0)	89 (42.2)	84 (39.8)	
**Yes**	38 (18.0)	16 (7.6)	22 (10.4)	
Clinical stage				0.3
**I**	21 (10.0)	14 (6.6)	7 (3.3)	
**II**	75 (35.5)	37 (17.5)	38 (18.0)	
**III**	78 (37.0)	39 (18.5)	39 (18.5)	
**IV**	37 (17.5)	15 (7.1)	22 (10.4)	
Pathological type				0.98
**IDC**	201 (95.3)	100 (47.4)	101 (47.9)	
**no IDC**	10 (4.7)	5 (2.4)	5 (2.4)	
Histological grade				0.86
**I–II**	126 (59.7)	65 (30.8)	61 (28.9)	
**III**	85 (40.3)	40 (19.0)	45 (21.3)	
Primary surgical style				0.16
**BCS**	21 (10.0)	14 (6.6)	7 (3.3)	
**MRM**	153 (72.5)	76 (36.0)	77 (36.5)	
**Only biopsy**	37 (17.5)	15 (7.1)	22 (10.4)	
NAST and AST				0.34
**Unknown**	4 (1.9)	3 (1.4)	1 (0.5)	
**No**	45 (21.3)	19 (9.0)	26 (12.3)	
**Yes**	162 (76.8)	83 (39.3)	79 (37.4)	
At initial BM progression				
Age				0.81
**<60 years**	139 (65.9)	70 (33.2)	69 (32.7)	
**≥60 years**	72 (34.1)	35 (16.6)	37 (17.5)	
KPS				0.22
**60**	52 (24.6)	31 (14.7)	21 (10.0)	
**70–80**	136 (64.5)	62 (29.4)	74 (35.1)	
**90**	23 (10.9)	12 (5.7)	11 (5.2)	
Infratentorial				0.42
**No**	82 (38.9)	44 (20.9)	38 (18.0)	
**Yes**	129 (61.1)	61 (28.9)	68 (32.2)	
Number of BM lesions				0.2
**1**	46 (21.8)	21 (10.0)	25 (11.8)	
**2–4**	108 (51.2)	50 (23.7)	58 (27.5)	
**>4**	57 (27.0)	34 (16.1)	23 (10.9)	
Clinical symptom				0.48
**Absence**	38 (18.0)	19 (9.0)	19 (9.0)	
**Presence**	173 (82.0)	86 (40.8)	87 (41.2)	
Meningeal metastases				0.34
**No**	190 (90.0)	93 (44.1)	97 (46)	
**Yes**	21 (14.7)	12 (5.7)	9 (4.3)	
**Leptomeningeal**	16 (7.6)	8 (3.8)	8 (3.8)	
**Dural**	5 (2.4)	4 (1.9)	1 (0.5)	
mBreast-GPA score				0.7
**Meningeal metastases and 0-1**	83 (39.3)	39 (18.5)	44 (20.9)	
**1.5–2**	80 (37.9)	42 (19.9)	38 (18.0)	
**2.5–3**	44 (20.9)	21 (10.0)	23 (10.9)	
**3.5**	4 (1.9)	3 (1.4)	1 (0.5)	
Extracranial metastatic sites				0.56
**No**	35 (16.6)	19 (9.0)	16 (7.6)	
**Yes**	179 (83.4)	88 (40.8)	92 (42.7)	
**Bone**	77 (36.0)	40 (18.5)	39 (17.5)	
**Liver**	44 (20.9)	21 (10.0)	23 (10.9)	
**Lung**	90 (42.6)	48 (22.7)	42 (20.2)	
**Lymph**	56 (26.5)	28 (13.2)	28 (13.3)	
**Others**	24 (11.4)	9 (4.3)	15 (0.7)	

MDT, multidisciplinary team; A, Luminal A; B(HER2−), Luminal B (HER2−); B(HER2+), Luminal B (HER2+); HER‐2+, HER‐2 overexpression; TN, triple‐negative; ER/PR, estrogen or progesterone receptor; IDC, invasive ductal carcinoma; BCS, breast-conserving therapy; MRM, modified radical mastectomy; NAST, neo‐adjuvant systemic therapy; AST, adjuvant systemic therapy; BM, brain metastasis; KPS, Karnofsky performance status; mBreast-GPA score, modified Graded Prognostic Assessment score.

**Table 2:** Disease status and treatment characteristics of two groups after initial BM or DM.

Characteristics	All	MDT	Non-MDT	*p-*value
	*n* = 211 (%)	*n* = 105 (%)	*n* = 106(%)	
Patient disease status throughout the course of disease				
Symptomatic tumor progression				0.04
No	175 (82.9)	81 (38.4)	94 (44.5)	
Yes	36 (17.1)	24 (11.2)	12 (5.7)	
Meningeal metastases				0.02
No	168 (80.8)	74 (35.6)	94 (45.2)	
Leptomeningeal	33 (15.9)	24 (11.5)	9 (4.3)	
Dural	7 (3.4)	6 (2.9)	1 (0.5)	
Lines of BM progression				<0.001
1	153 (72.6)	59 (27.9)	94 (44.7)	
2	44 (20.7)	33 (15.4)	11 (5.3)	
3	8 (3.8)	8 (3.8)	0 (0)	
4	6 (2.9)	6 (2.9)	0 (0)	
Posterior treatment after initial BM or DM				
Courses of intracranial RT				<0.001
0	26 (12.3)	10 (4.8)	16 (7.7)	
1	139 (65.8)	55 (26.4)	84 (39.4)	
≥2	46 (21.8)	40 (18.9)	6 (2.9)	
Intracranial RT at initial BM				<0.001
No RT	26 (12.3)	10 (4.7)	16 (7.6)	
WBRT only	121 (57.3)	47 (22.3)	74 (35.1)	
SRS/WBRT-HA	64 (30.3)	48 (22.7)	16 (35.1)	
Summary of intracranial RT techniques				<0.001
WBRT only	106 (50)	34 (16.3)	72 (33.7)	
SRS/WBRT-HA	79 (37.4)	61 (28.8)	18 (8.6)	
WBRT-HA only	8 (3.8)	5 (2.4)	3 (1.4)	
SRS/fSRS	71 (33.6)	56 (26.4)	15 (7.2)	
Posterior systemic therapy lines after initial BM				<0.01
(median, IQR)		1 (IQR: 1–2)	1 (IQR: 0–2)	
Prospective clinical research				<0.001
No	171 (80.8)	73 (34.6)	98 (46.2)	
Yes	40 (19.2)	32 (15.4)	8 (3.8)	
New drugs				<0.001
No	160 (76)	62 (29.3)	98 (46.6)	
Yes	51 (24)	44 (20.7)	7 (3.4)	
Re-biopsy				0.677
No	103 (48.1)	53 (25)	50 (23.1)	
Yes	108 (51.9)	52 (25)	56 (26.9)	
HER2+ status	*n* = 86 (40.4)	*n* = 48 (22.6)	*n* = 38 (17.8)	
Trastuzumab				0.231
No	7 (3.4)	2 (1.0)	5 (2.4)	
Yes	79 (37)	46 (21.6)	33 (15.4)	
Pertuzumab				0.001
No	68 (32.7)	32 (15.4)	36 (17.3)	
Yes	17 (7.7)	16 (7.2)	1 (0.5)	
Tykerb and Pyrotinib Maleate				0.003
No	33 (15.9)	15 (7.2)	18 (8.7)	
Tykerb	22 (10.6)	8 (3.8)	14 (6.7)	
Pyrotinib Maleate	25 (11.5)	21 (9.6)	4 (1.9)	
Both	5 (2.4)	4 (1.9)	1 (0.5)	

MDT, multidisciplinary team; RT, radiotherapy; WBRT, whole-brain radiation therapy; WBRT-HA, WBRT with hippocampal avoidance; SRS, stereotactic radiosurgery; fSRT, fractionated stereotactic radiotherapy; BM, brain metastasis; DM, distant metastasis; IQR, interquartile range.

**Table 3:** Multivariate regression analyses of receiving courses of intracranial RT.

Characteristics	Multivariate analysis	*p*
	odds ratio (95%CI)	
Molecular subtype		
TN	1	
HER2+	0.1 (0.01–1.1)	0.058
B(HER2+)	0.4 (0.1–1.4)	0.151
B(HER2−)	0.4 (0.1–1.51)	0.178
A	0.3 (0.1–0.9)	0.044
No MDT	1	
MDT	9.1 (2.9–21.3)	<0.001
Primary surgery		
Only biopsy	1	
BCS	2.0 (0.4–11.3)	0.43
MRM	0.4 (0.1–1.2)	0.083
Intracranial RT at initial BM		
SRS/WBRT-HA	1	
WBRT only	0.2 (0.1–0.6)	0.002
New drugs		
No	0.7 (0.2–2.8)	0.585
Yes	1	
Prospective clinical trial		
No	0.6 (0.2–2.0)	0.457
Yes	1	
Re-biopsy		0.07
No	0.4 (0.2–1.1)	
Yes	1	

RT, radiotherapy; MDT, multidisciplinary team; A, Luminal A; B(HER2−), Luminal B (HER2−); B(HER2+), Luminal B (HER2+); HER‐2+, HER‐2 overexpression; TN, triple‐negative; BCS, breast-conserving therapy; MRM, modified radical mastectomy; BM, brain metastasis; WBRT, whole-brain radiation therapy; WBRT-HA, WBRT with hippocampal avoidance; SRS, stereotactic radiosurgery; fSRT, fractionated stereotactic radiotherapy.

**Table 4:** Multivariate regression analyses associated with radiotherapeutic techniques (SRS/WBRT-HA vs. WBRT only).

Characteristics	Multivariate analysis	*p-*value
	odds ratio (95% CI)	
**No MDT**	1	
**MDT**	7.0 (3.3–14.7)	<0.001
Primary surgery		
Only biopsy	1	
BCS	1.2 (0.3–5.0)	0.831
MRM	0.5 (0.2–1.3)	0.163
**Infratentorial**		
No	1.7 (0.9–3.5)	0.116
Yes	1	
Modified Breast-GPA score		
≥2.5	1	
Meningeal metastases and 0–1	0.5 (0.2–1.8)	0.129
1.5–2	1.1 (0.5–2.7)	0.803
New drugs		
No	0.7 (0.2–2.6)	0.61
Yes	1	
Prospective clinical trial		
No	0.7 (0.3–1.8)	0.447
Yes	1	
**Re-biopsy**		0.845
No	1.0 (0.5–2.1)	
Yes	1	

RT, radiotherapy; MDT, multidisciplinary team; WBRT, whole-brain radiation therapy; WBRT-HA, WBRT with hippocampal avoidance; SRS, stereotactic radiosurgery; fSRT, fractionated stereotactic radiotherapy; BCS, breast-conserving therapy; MRM, modified radical mastectomy.

**Table 5:** Multivariate regression analyses associated with participation in prospective clinical trials.

Characteristics	Multivariate analysis	*p*
	odds ratio (95% CI)	
No MDT	1	
MDT	4.7 (1.2–11.9)	0.001
NAST and AST		
No/Unknown	0.53 (0.2–1.5)	0.228
Yes	1	
Posterior systemic therapy lines after initial BM		
0	0.3 (0.07–1.3)	0.119
1	0.6 (0.3–1.5)	0.343
≥2	1	
HER-2 status		
Negative	1	
Positive	2.3 (1.1–4.6)	0.039
Clinical symptom		
Absence	6.2 (0.9–16.5)	0.068
Presence	1	
Re-biopsy		
No	2.3 (0.9–5.1)	0.053
Yes	1	
Grade		
I-II	2.4 (0.9–6.1)	0.054
III	1	

RT, radiotherapy; MDT, multidisciplinary team; NAST, neo‐adjuvant systemic therapy; AST, adjuvant systemic therapy; BM, brain metastasis.

## Results

### Baseline patient characteristics at the first presentation and the initial BM

**Figure 1:**
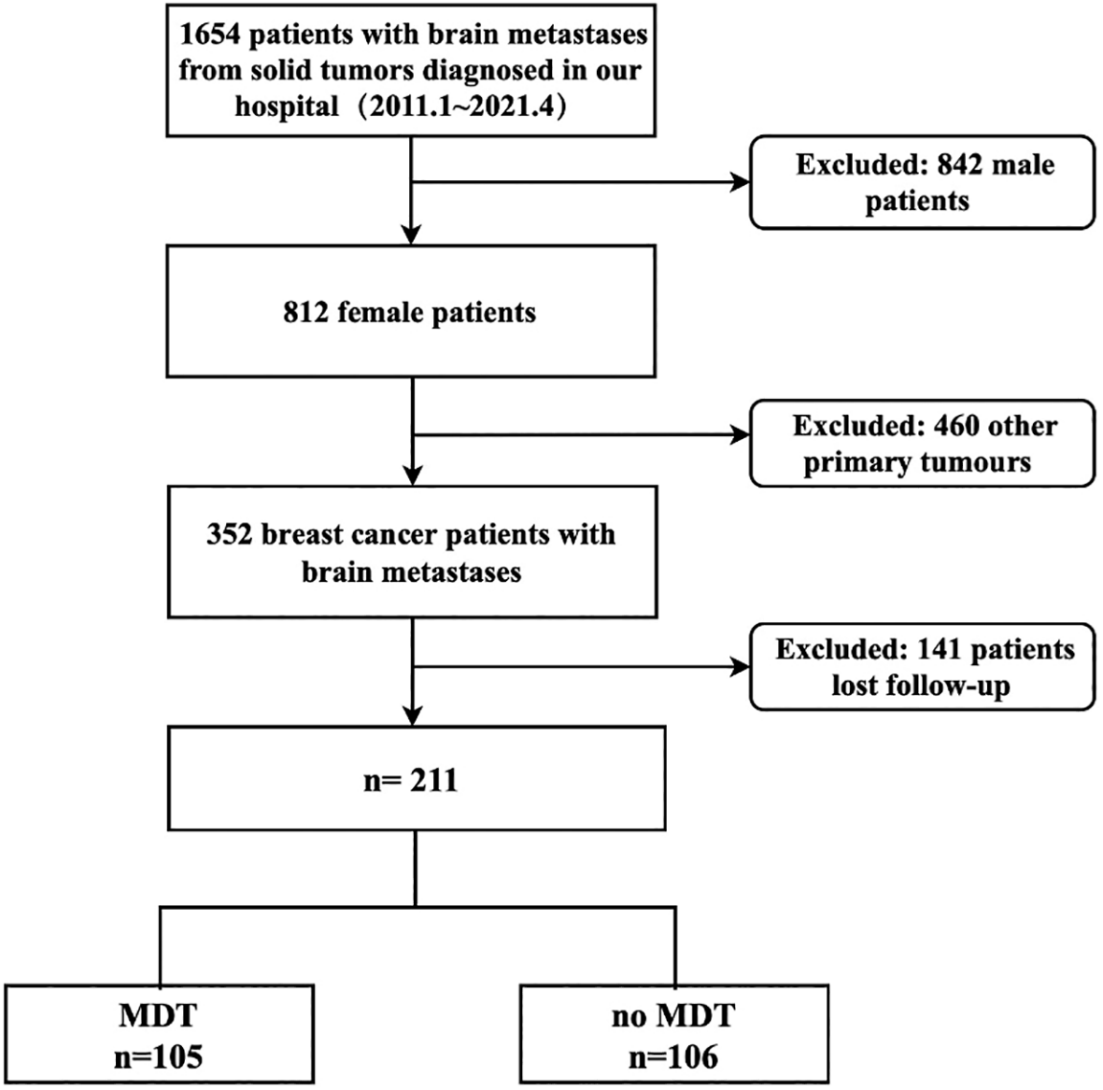
Flowchart of enrollment.

A total of 211 female patients diagnosed with BCBM with follow-up information were eligible for inclusion in the current study (see **Figure 1**). Among these patients, 105 (49.8%) attended at least one MDT discussion while 106 (50.2%) did not. At baseline, the clinicopathological, treatment, and characteristics at the first presentation and the initial BM were generally balanced between the two groups (**Table 1**). There were 35.5 (16.6%) patients and 37 (17.5%) patients aged older than 60 years in the MDT and no-MDT cohorts, respectively. Patients with Luminal A, Luminal B, triple‐negative, and HER2 overexpression were 10 (4.7%), 49 (23.2%), 25 (11.8%), and 21 (10.0%) in the MDT group, and 13 (6.2%), 35 (16.6%), 22 (10.4%), and 36 (17.1%) in the no-MDT group, respectively.

### MDT and “diagnosis updated”

**Figure 2:**
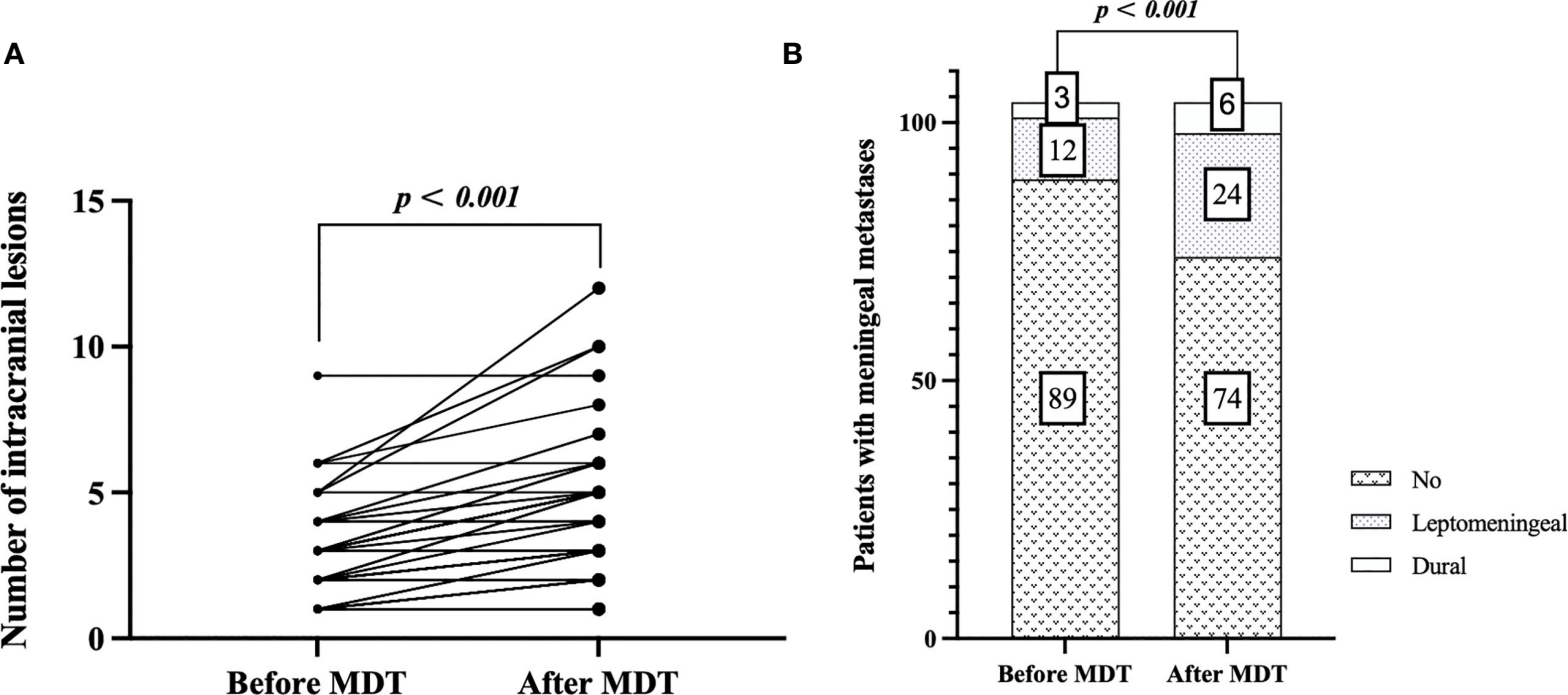
Estimation plot of intracranial “diagnosis updated”: comparison of number of intracranial lesions before and after MDT discussion at initial brain metastasis **(A)**, and comparison of meningeal metastases before and after MDT discussion throughout the course of disease **(B)**.

Among the MDT group, all patients with initial BM and a proportion of patients with recurrent brain metastases participated in the MDT meeting. In the MDT group (*n* = 105), 56 cases (53.3%) were documented as intracranial “diagnosis updated” at initial BM. Six cases (5.7%) were revised from 0 to 1 lesion, 4 (3.8%) from “single lesion” to “limited metastases (2–4 lesions)”, and 25 (23.8%) from “limited metastases” to multiple metastasis” (>4 lesions). The median number of intracranial BM lesions in patients before and after MDT discussion were 3 [interquartile range (IQR): 1–4] and 3 (IQR: 2–5), respectively (*p <* 0.001) (see **Figure 2A**). Throughout the whole course of disease of BM, 14 cases (13.4%) were documented as extracranial “diagnosis updated”, including new metastatic lesions in the skull (6.7%), adrenal gland (5.8%), and musculus ocularis (1.0%). Before MDT, 89 patients (84.8%) were diagnosed with no meningeal metastases, 12 patients (11.4%) were diagnosed with leptomeningeal metastases, and 3 patients (2.9%) were diagnosed with dural metastases. After MDT, another 12 cases (11.4%) were revised from no meningeal metastases to leptomeningeal metastases, and 3 (2.9%) were revised from no meningeal metastases to dural metastases (see **Figure 2B**).

These updated diagnoses led to revision of the therapeutic decision, including a switch from WBRT-HA to WBRT in four cases (3.8%) following the discovery of a new lesion close to the hippocampus and a switch to WBRT from SRS/fSRT in 10 patients (9.6%) following newly found multifocal or meningeal metastases.

### Disease status throughout the whole course of disease

During the disease course, patients in the MDT group were recorded a higher proportion of meningeal metastases (14.3% vs. 4.8%, *p* = 0.02), symptomatic tumor progression such as moderate amount of malignant pericardial/pleural effusion or pathologic fracture (11.2% vs. 5.7%, *p* = 0.04), and an increased number of occurrences of BM progression (*p <* 0.05) (**Table 2**).

### Influence of MDT on therapeutic strategy

Treatment information associated with local intracranial or systematic therapies after the initial BM or distant metastases (DM) were collected. As shown in **Table 2**, the number of intracranial RT courses, techniques of intracranial RT, enrollment in clinical trials, utilization of new agents, and number of posterior-line systemic therapy after initial BM exhibited significant differences between the two groups (all *p <* 0.05, **Table 2**).

Among the nine patients who underwent surgical resection followed by postsurgical intracranial RT, five were in the MDT group and four were in the non-MDT group. A total of 185 patients (87.6%) received intracranial RT. The MDT group had a higher proportion of patients receiving ≥2 courses of intracranial RT compared to the non-MDT group (18.9% vs. 2.9%, *p <* 0.001, **Table 2**). Factors such as primary surgery style, re-biopsy after initial DM or BM, and intracranial RT techniques at initial BM were associated with the number of courses of intracranial RT (all *p* < 0.05, **Supplementary Table 2**). On multivariable ordinal logistic regression analysis, attending MDT [OR 9.1, 95% confidence interval (CI) 2.9–21.3, *p* < 0.001] was associated with an increased course of intracranial RT (**Table 3**). Patients treated with WBRT at initial BM had approximately 80% lower odds of receiving repeat intracranial RT compared to those treated with novel RT techniques such as WBRT-HA or SRS/fSRS (OR 0.2, 95% CI 0.1–0.6,  *p* = 0.002). Molecular subtype showed no statistical significance in univariate analysis, but luminal A type showed a lower proportion of repeat RT compared to triple-negative type on multivariate analysis (OR 0.3, 95% CI 0.1–0.9, *p* = 0.044) (**Table 3**).

Patients in the MDT group had a higher likelihood of being treated with novel RT techniques such as WBRT-HA or SRS/fSRS compared to the non-MDT group (28.8% vs. 8.7%, *p <* 0.001). Univariate analysis also revealed that factors such as no infratentorial involvement and primary surgery style were significantly associated with the use of novel RT techniques (all *p <* 0.05, **Supplementary Table 3**). On multivariable analysis, attending MDT (OR 7.0, 95% CI 3.3–14.7, *p <* 0.001) was the only independent factor associated with novel RT technique use (see **Table 4**).

In terms of systemic therapy, the MDT group had a higher likelihood to undergo multiple lines of posterior systemic therapy compared to the non-MDT group [no-MDT vs. MDT (median, IQR): 1 (IQR: 0–2) vs. 1 (IQR: 1–2), *p* < 0.001]. A total of 51 (24.1%) patients received treatment with new anti-cancer agents. The MDT group had a higher opportunity to receive new agents for posterior systemic therapy lines after initial BM than non-MDT patients (20.7% vs. 3.4%, *p <* 0.001). Among 40 (19.2%) patients enrolled in clinical trials, 26 (65%) were treated with new agents. In univariate analysis, MDT patients had a higher chance of participating in clinical trials compared to non-MDT patients (15.2% vs. 3.8%, *p <* 0.001). Other factors, such as HER-2 positive status, grade I–II at presentation, receiving neo-adjuvant systematic therapy (NAST) and adjuvant systematic therapy (AST), clinical symptom at initial BM, and increased posterior line systemic therapy after initial BM were associated with PCT participation (**Supplementary Table 4**). Multivariate analysis showed that attending MDT (OR 4.7, 95% CI 1.2–11.9, *p* = 0.001) and HER-2-positive status **(**OR 2.3, 95% CI 1.1–4.6, *p* = 0.039) were independently associated with PCT participation (**Table 5**).

### Analysis of disease outcomes

**Figure 3:**
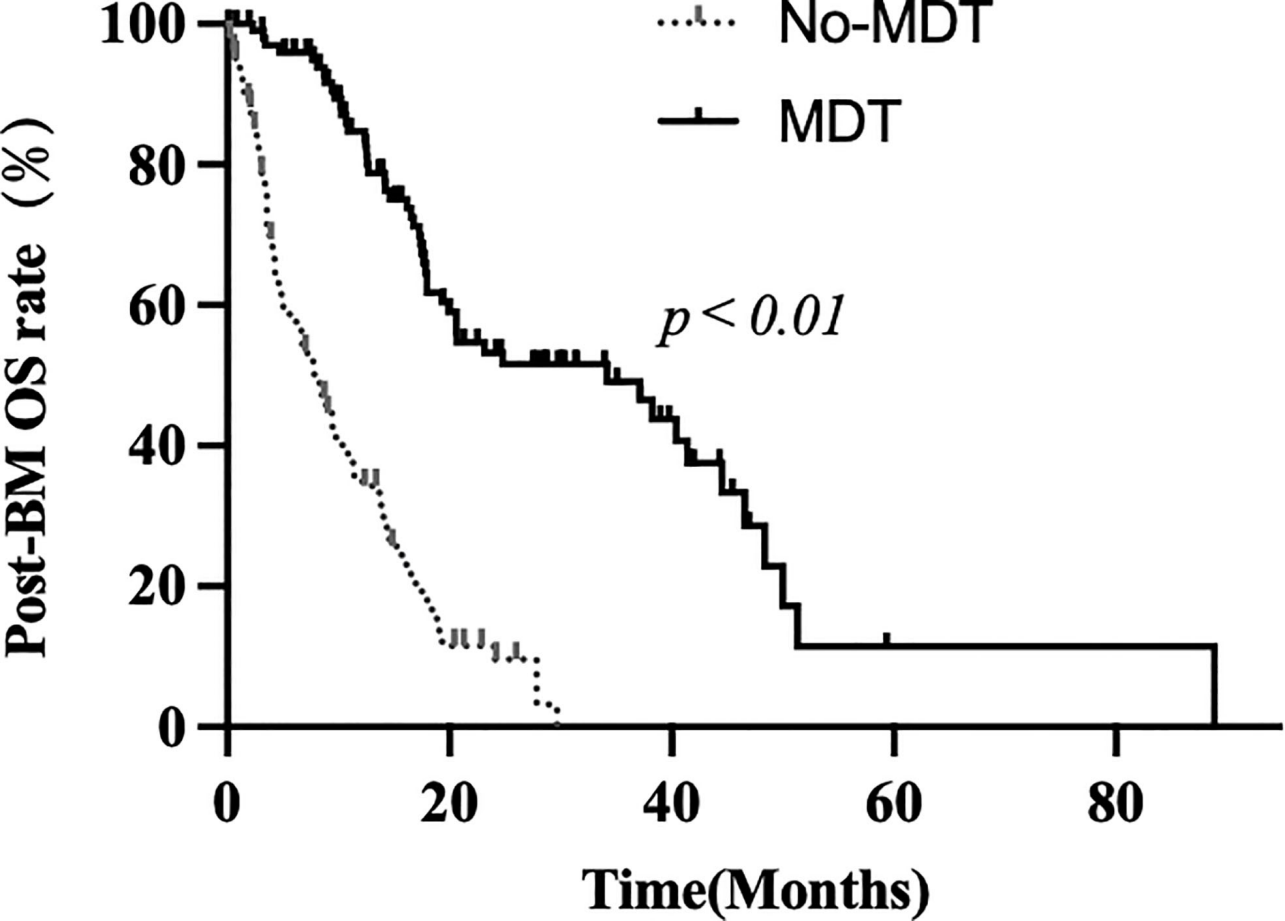
Kaplan–Meier analysis of MDT and non-MDT: post-brain metastases overall survival.

After a median follow-up time of 50.2 months, a total of 134 death events were recorded, with 84 in the non-MDT group and 50 in the MDT group. The post-BM OS was 15.5 ± 1.2 months in the entire cohort, with 34.2 ± 8.4 months in the MDT group and 8.2 ± 1.1 months in the non-MDT group (*p* < 0.01, **Figure 3**).

## Discussion

This research included 211 patients diagnosed as BCBM who received intracranial RT and posterior line systemic therapy after MDT discussion or not. We compared the pattern of treatment in patients who participated in MDT and those who did not participate, and thus analyzed the impact of MDTs on BCBM patients in a public health center. The findings of this research highlighted that the increased chances of receiving repeat intracranial RT, innovative RT techniques, and PCT participation significantly correlate with MDT discussion. In addition, MDT discussions could also play a valuable role of “diagnosis upgraded” in the diagnostic process. These improvements in treatment patterns ultimately led to better survival outcomes for patients involved in MDT discussions. To the best of our knowledge, it is the first study to provide direct evidence supporting the beneficial effects of MDTs in improving treatment patterns for BCBM patients.

MDT models for solid tumors with BM have been recommended as a standard strategy by the National Comprehensive Cancer Network (NCCN) guidelines as far back as 2011 (11). However, there are notable differences in the structures and functions of MDTs across countries and regions (6, 12). In the United Kingdom, MDT discussions are mandatory for all BC patients (7), whereas they are only mandatory in less than half of the countries in Asia (6). At our health center, the BC MDT system is routinely utilized for addressing surgery and (neo)adjuvant therapy for early BC cases (13). In our current research, BC patients have nearly a 50% chance of participating in MDT discussion after developing BM, particularly those with complex conditions such as tumor-related complications, recurrent BM, or meningeal metastases throughout the course of BM disease. One role of MDT is to provide a multidisciplinary combination of therapeutic interventions. Previous studies have also supported the positive impact of MDTs on improved medical and palliative care (14). In our study, out of the 36 (17.3%) patients with symptomatic tumor progression, 24 (11.5%) patients were recommended for MDT discussions to alleviate their symptoms. BC is the second most common malignant tumor associated with various organ-specific metastases, both intra- and extracranially (15–17). These complications often contribute to poor prognosis (18–20) and serve as common reasons for MDT referrals (21, 22). Another important role of MDTs is to facilitate effective and efficient communication among different specialists (23), shortening the time required for differential diagnosis and therapeutic decision-making (24, 25). In our current research, although only the intracranial lesions at the time of initial BM were recorded, it was found that 53.3% of patients experienced upgrades in their intracranial diagnoses following MDT discussions. Throughout the entire course of BM, 14.3% of patients were diagnosed with meningeal metastases, resulting in corresponding modifications to their therapeutic strategies in 15.7% of cases. These findings confirm our hypothesis that MDTs provide efficient diagnostic and therapeutic interventions.

Over the past decades, the therapeutic approaches for BCBM have greatly evolved (26). Systemic therapy has become increasingly important, especially for HER2-positive disease. The novel anticancer agents such as monoclonal antibodies, tyrosine kinase inhibitors, and ADCs targeting the HER2 receptor have expanded treatment options. CDK4/6 inhibitors for hormone receptor-positive disease and immunotherapy for triple-negative disease have also made a significant impact. However, a part of these therapies was less available before they were included by national healthcare since 2019. Yang reported that 40.5% of HER2-positive patients with BM did not receive anti-HER2 therapy between 2003 and 2015 (19). In a previous study from our center, Ou et al. reported that anti-HER2 agents were used in only 30.8% of BMs from HER2-positive BC between 2009 and 2017 (27). In our current research, by the end of 2021, 20.7% of patients in the MDT group had received at least one of the new agents, which was only 3.4% in the non-MDT group (*p* < 0.001). MDT patients also had higher rates of receiving multiple lines of systemic therapy after developing BM compared to those without MDT involvement (*p* < 0.001). In addition, MDT patients were also more likely to participate in PCT, especially patients with HER2-positive disease. Patients in the MDT group have nearly four times higher odds of participating in receiving PCT than those in the non-MDT group. This is in line with previous literature that show that MDT could increase in the proportion of patients being considered for clinical trials (6), supporting the recommendations of guidelines such as those of the American Society of Clinical Oncology (ASCO), the Society for Neuro-Oncology (SNO), and the American Society for Radiation Oncology (ASTRO) (8), and Neuro-Oncology (EANO)–European Society for Medical Oncology (ESMO) Guidelines, which encourage eligible patients to participate in clinical research and highlight the benefits of MDT collaboration in enhancing the overall quality of systemic treatment for BCBM.

When systemic therapy contributes to extracranial disease control, the need for better intracranial disease control with less toxicities is increasing. According to EANO–ESMO Clinical Practice Guidelines (28), surgical resection could rapidly relieve symptoms of cerebral edema and hydrocephalus. However, in our current research, only a few patients underwent surgical resection for extracranial metastases. Instead, more than 85% of patients received intracranial RT. SRS and WBRT-HA were high-precision RT approaches that cause less cognitive deterioration compared to conventional WBRT (29–31). A previous radiologic study from our team also found a low rate of hippocampal involvement in newly diagnosed BMs from BC and lung cancer (32). The indication of SRS/fSRT depends on the anatomical location and size of a single metastasis and is less limited by the number of metastases than before. SRS/fSRT has shown comparable therapeutic outcomes in patients with 4 to 10 brain metastases compared to those with 1–3 metastases (33, 34). In our study, patients in the MDT group underwent a more comprehensive review of radiological images. As a result, 53.3% of patients had their intracranial “diagnosis updated” regarding the number of lesions (*p <* 0.05). Although some patients had their RT strategy modified after the updated diagnosis, MDT patients were approximately 6.0 times more likely to undergo novel RT techniques compared to non-MDT patients (OR 7.0, 95% CI 3.3–14.7, *p <* 0.001). Following SRS/fSRT alone, local or distant brain failures have been reported in 25% to 50% of patients within 1 year (35, 36). The salvage strategy typically involves WBRT or a repeated course of SRS/fSRT. Moreau et al. demonstrated that implementing a second SRS required careful dose and volume limitations to ensure favorable rates of local control and acceptable toxicities (37). In our study, one factor influencing repeat intracranial RT was the previous technique used for the initial BM. Patients who have previously received novel technique had four times higher odds of repeat intracranial RT compared to those who have not (WBRT vs. WBRT-HA or SRS/fSRS: OR 0.2, 95% CI 0.1–0.6,  *p* = 0.002). Another important factor was attending MDT. MDT patients had eight times higher odds of undergoing repeat intracranial RT (OR 9.1, 95% CI 2.9–21.3, *p* < 0.001) when there was progression of BM. This was due to the communication platform facilitating a better differential diagnosis of disease progression or necrosis through the evaluation of treatment planning and MRI in the MDT, particularly for patients who had received previous intracranial RT. This helps guide the decision-making regarding salvage WBRT or a repeated SRS strategy.

In summary, MDT plays a crucial role in promoting the practical application of local and systemic treatment patterns. Huang et al. discovered that patients who received repeat RT to control intracranial progression experienced greater clinical benefits in terms of progression-free survival, compared to those with uncontrolled intracranial lesions (7.7 vs. 4.6 months, *p* = 0.009) (38), suggesting a potential delay in death through repeat RT. Walker et al. demonstrated that patients receiving SRS had longer OS than those receiving WBRT (HR 0.633 [0.535–0.750]; *p* < 0.001) (39). In our current research, owing to more comprehensive evaluation of the intracranial and extracranial metastatic burden, increased utilization of novel anticancer agents, and higher application rate of precise modern radiotherapeutic techniques, all these treatment patterns improved by MDT contributed to enhanced OS outcomes for patients after BM (34.2 ± 8.4 vs. 8.2 ± 1.1 months, *p* < 0.01).

As a retrospective study, our study has some inherent limitations, such as inevitable selection bias. The relatively limited sample size prevents us from further discussing the personalized treatment plans and performing recognition analysis. In addition, our research does not provide detailed descriptions of toxicity, adverse reactions, or the timing of RT interventions. Nevertheless, our data can serve as encouragement for physicians to organize and refer BM patients to MDTs.

## Conclusion

Patients with complex conditions such as tumor-related complications, meningeal metastases, or repeated BM progressions are more likely to be referred to MDT discussion. Our study suggests that MDT for BCBM patients improves the pattern of care including both intracranial therapeutic decision and systemic therapy choices, resulting in benefits of OS after BM. Our finding encourages the recommendations of BMBC patients to be presented to an MDT not only at first diagnosis of BM but also at the time of subsequent progresses.

## Data availability statement

The original contributions presented in the study are included in the article/**Supplementary Material**. Further inquiries can be directed to the corresponding author.

## Author contributions

FX (First Author): Conceptualization, Methodology, Software, Investigation, Formal Analysis, Writing - Original Draft. DO: Data Curation, Formal Analysis. WQ: Writing - Review & Editing, Methodology. SW: Methodology. YH: Data Curation. GC: Data Curation. LC: Data Curation. CX: Data Curation. JC (Corresponding Author): Conceptualization, Funding Acquisition, Resources, Supervision, Writing - Original Draft, Writing - Review & Editing. All authors contributed to the article and approved the submitted version.

## References

[1] Siegel RL Miller KD Jemal A. Cancer statistics, 2019. CA Cancer J Clin (2019) 69(1):7–34. doi: 10.3322/caac.21551 30620402

[2] Sung H Ferlay J Siegel RL Laversanne M Soerjomataram I Jemal A. Global cancer statistics 2020: GLOBOCAN estimates of incidence and mortality worldwide for 36 cancers in 185 countries. CA: A Cancer J Clin (2021) 71(3):209–49. doi: 10.3322/caac.21660 33538338

[3] Lee YT. Breast carcinoma: pattern of metastasis at autopsy. J Surg Oncol (1983) 23(3):175–80. doi: 10.1002/jso.2930230311 6345937

[4] Jung SY Rosenzweig M Sereika SM Linkov F Brufsky A Weissfeld JL. Factors associated with mortality after breast cancer metastasis. Cancer Causes Control (2012) 23(1):103–12. doi: 10.1007/s10552-011-9859-8 22037907

[5] Zhao S Qi W Chen J. Role of a multidisciplinary team in administering radiotherapy for esophageal cancer. BMC Cancer (2020) 20(1):974. doi: 10.1186/s12885-020-07467-z 33032547 PMC7545841

[6] Saini KS Taylor C Ramirez AJ Palmieri C Gunnarsson U Schmoll HJ. Role of the multidisciplinary team in breast cancer management: results from a large international survey involving 39 countries. Ann Oncol (2012) 23(4):853–9. doi: 10.1093/annonc/mdr352 21821551

[7] English R Metcalfe C Day J Rayter Z Blazeby JM. A prospective analysis of implementation of multi-disciplinary team decisions in breast cancer. Breast J (2012) 18(5):459–63. doi: 10.1111/j.1524-4741.2012.01270.x 22776015

[8] Vogelbaum MA Brown PD Messersmith H Brastianos PK Burri S Cahill D. Treatment for brain metastases: ASCO-SNO-ASTRO guideline. J Clin Oncol (2022) 40(5):492–516. doi: 10.1200/JCO.21.02314 34932393

[9] Li Y Li Q Mo H Guan X Lin S Wang Z. Incidence, risk factors and survival of patients with brain metastases at initial metastatic breast cancer diagnosis in China. Breast (2021) 55:30–6. doi: 10.1016/j.breast.2020.11.021 PMC7736978 33310633

[10] Sperduto PW Mesko S Li J Cagney D Aizer A Lin NU. Beyond an updated graded prognostic assessment (Breast GPA): A prognostic index and trends in treatment and survival in breast cancer brain metastases from 1985 to today. Int J Radiat Oncol Biol Phys (2020) 107(2):334–43. doi: 10.1016/j.ijrobp.2020.01.051 PMC7276246 32084525

[11] Brem SS Bierman PJ Brem H Butowski N Chamberlain MC Chiocca EA. Central nervous system cancers. J Natl Compr Cancer Network J Natl Compr Canc Netw (2011) 9(4):352–400. doi: 10.6004/jnccn.2011.0036 21464144

[12] Anderson BO Yip CH Smith RA Shyyan R Sener SF Eniu A. Guideline implementation for breast healthcare in low-income and middle-income countries: overview of the Breast Health Global Initiative Global Summit 2007. Cancer (2008) 113(8 Suppl):2221–43. doi: 10.1002/cncr.23844 18816619

[13] Yang X Huang J Zhu X Shen K Zhu J Chen X. Compliance with multidisciplinary team recommendations and disease outcomes in early breast cancer patients: An analysis of 4501 consecutive patients. Breast (2020) 52:135–45. doi: 10.1016/j.breast.2020.05.008 PMC7375553 32512360

[14] Chirgwin J Craike M Gray C Watty K Mileshkin L Livingston PM. Does multidisciplinary care enhance the management of advanced breast cancer?: evaluation of advanced breast cancer multidisciplinary team meetings. J Oncol Pract (2010) 6(6):294–300. doi: 10.1200/JOP.2010.000017 21358959 PMC2988663

[15] Penz E Watt KN Hergott CA Rahman NM Psallidas I. Management of Malignant pleural effusion: challenges and solutions. Cancer Manag Res (2017) 9:229–41. doi: 10.2147/CMAR.S95663 PMC5491570 28694705

[16] Song MJ Jo U Jeong JS Cho KJ Gong G Cho YM. Clinico-cytopathologic analysis of 574 Pericardial Effusion Specimens: Application of the international system for reporting serous fluid cytopathology (ISRSFC) and long-term clinical follow-up. Cancer Med (2021) 10(24):8899–908. doi: 10.1002/cam4.4408 PMC8683522 34747147

[17] Li S Peng Y Weinhandl ED Blaes AH Cetin K Chia VM. Estimated number of prevalent cases of metastatic bone disease in the US adult population. Clin Epidemiol (2012) 4:87–93. doi: 10.2147/CLEP.S28339 22570568 PMC3345874

[18] Gornik HL Gerhard-Herman M Beckman JA. Abnormal cytology predicts poor prognosis in cancer patients with pericardial effusion. J Clin Oncol (2005) 23(22):5211–6. doi: 10.1200/JCO.2005.00.745 16051963

[19] Yang Y Du J Wang YS Kang HY Zhai K Shi HZ. Prognostic impact of pleural effusion in patients with Malignancy: A systematic review and meta-analysis. Clin Transl Sci (2022) 15(6):1340–54. doi: 10.1111/cts.13260 PMC9199884 35212454

[20] Katagiri H Takahashi M Wakai K Sugiura H Kataoka T Nakanishi K. Prognostic factors and a scoring system for patients with skeletal metastasis. J Bone Joint Surg Br (2005) 87(5):698–703. doi: 10.1302/0301-620X.87B5.15185 15855375

[21] Refaat MM Katz WE. Neoplastic pericardial effusion. Clin Cardiol (2011) 34(10):593–8. doi: 10.1002/clc.20936 PMC6652358 21928406

[22] Banka R George V Rahman NM. Multidisciplinary approaches to the management of Malignant pleural effusions: a guide for the clinician. Expert Rev Respir Med (2020) 14(10):1009–18. doi: 10.1080/17476348.2020.1793672 32634337

[23] Ruhstaller T Roe H Thürlimann B Nicoll JJ. The multidisciplinary meeting: An indispensable aid to communication between different specialities. Eur J Cancer (2006) 42(15):2459–62. doi: 10.1016/j.ejca.2006.03.034 16934974

[24] Gabel M Hilton NE Nathanson SD. Multidisciplinary breast cancer clinics. Do they work? Cancer (1997) 79(12):2380–4. doi: 10.1002/(SICI)1097-0142(19970615)79:12<2380::AID-CNCR12>3.0.CO;2-N 9191526

[25] Jung J Tailor J Dalton E Glancz LJ Roach J Zakaria R. Management evaluation of metastasis in the brain (MEMBRAIN)-a United Kingdom and Ireland prospective, multicenter observational study. Neurooncol Pract (2020) 7(3):344–55. doi: 10.1093/nop/npz063 PMC7274191 32537183

[26] Sammons S Van Swearingen AED Chung C Anders CK. Advances in the management of breast cancer brain metastases. Neurooncol Adv (2021) 3(Suppl 5):v63–74. doi: 10.1093/noajnl/vdab119 PMC8633750 34859234

[27] Ou D Cao L Xu C Kirova Y Chen JY. Upfront brain radiotherapy may improve survival for unfavorable prognostic breast cancer brain metastasis patients with Breast-GPA 0-2.0. Breast J (2019) 25(6):1134–42. doi: 10.1111/tbj.13426 31286612

[28] Le Rhun E Guckenberger M Smits M Dummer R Bachelot T Sahm F. EANO–ESMO Clinical Practice Guidelines for diagnosis, treatment and follow-up of patients with brain metastasis from solid tumours☆. Ann Oncol (2021) 32(11):1332–47. doi: 10.1016/j.annonc.2021.07.016 34364998

[29] Brown PD Ballman KV Cerhan JH Anderson SK Carrero XW Whitton AC. Postoperative stereotactic radiosurgery compared with whole brain radiotherapy for resected metastatic brain disease (NCCTG N107C/CEC·3): a multicentre, randomised, controlled, phase 3 trial. Lancet Oncol (2017) 18(8):1049–60. doi: 10.1016/S1470-2045(17)30441-2 PMC5568757 28687377

[30] Brown PD Gondi V Pugh S Tome WA Wefel JS Armstrong TS. Hippocampal avoidance during whole-Brain radiotherapy plus memantine for patients with brain metastases: phase III trial NRG oncology CC001. J Clin Oncol (2020) 38(10):1019–29. doi: 10.1200/JCO.19.02767 PMC7106984 32058845

[31] Gondi V Tolakanahalli R Mehta MP Tewatia D Rowley H Kuo JS. Hippocampal-sparing whole-brain radiotherapy: a "how-to" technique using helical tomotherapy and linear accelerator-based intensity-modulated radiotherapy. Int J Radiat Oncol Biol Phys (2010) 78(4):1244–52. doi: 10.1016/j.ijrobp.2010.01.039 PMC2963699 20598457

[32] Han Y-M Cai G Chai W-M Xu C Cao L Ou D. Radiological distribution of brain metastases and its implication for the hippocampus avoidance in whole brain radiotherapy approach. Br J Radiol (2017) 90:20170099. doi: 10.1259/bjr.20170099 28830202 PMC5963386

[33] Yamamoto M Serizawa T Shuto T Akabane A Higuchi Y Kawagishi J. Stereotactic radiosurgery for patients with multiple brain metastases (JLGK0901): a multi-institutional prospective observational study. Lancet Oncol (2014) 15(4):387–95. doi: 10.1016/S1470-2045(14)70061-0 24621620

[34] Lo SS Sloan AE Machtay M. Stereotactic radiosurgery for more than four brain metastases. Lancet Oncol (2014) 15(4):362–3. doi: 10.1016/S1470-2045(14)70076-2 24621619

[35] Zindler JD Slotman BJ Lagerwaard FJ. Patterns of distant brain recurrences after radiosurgery alone for newly diagnosed brain metastases: implications for salvage therapy. Radiother Oncol (2014) 112(2):212–6. doi: 10.1016/j.radonc.2014.07.007 25082096

[36] Atkins KM Pashtan IM Bussière MR Kang KH Niemierko A Daly JE. Proton stereotactic radiosurgery for brain metastases: A single-Institution analysis of 370 patients. Int J Radiat Oncol Biol Phys (2018) 101(4):820–9. doi: 10.1016/j.ijrobp.2018.03.056 29976494

[37] Moreau J Khalil T Dupic G Chautard E Lemaire JJ Magnier F. Second course of stereotactic radiosurgery for locally recurrent brain metastases: Safety and efficacy. PLoS One (2018) 13(4):e0195608. doi: 10.1371/journal.pone.0195608 29621341 PMC5886580

[38] Huang Z Sun B Shen G Cha L Meng X Wang J. Brain metastasis reirradiation in patients with advanced breast cancer. J Radiat Res (2017) 58(1):142–8. doi: 10.1093/jrr/rrw087 PMC5321192 27707842

[39] Mainwaring W Bowers J Pham N Pezzi T Shukla M Bonnen M. Stereotactic radiosurgery versus whole brain radiation therapy: A propensity score analysis and predictors of care for patients with brain metastases from breast cancer. Clin Breast Cancer (2019) 19(2):e343–51. doi: 10.1016/j.clbc.2018.11.001 30527350

