# Supplementary material for: Impact of multidisciplinary team on the pattern of care for brain metastasis from breast cancer
Source: Front Oncol. 2023 Aug 16;13:1160802. doi: 10.3389/fonc.2023.1160802 (PMC10471195; doi:10.3389/fonc.2023.1160802)
Supplement: Supplementary file 1 [file Table_1.docx]

Supplementary Table 1. Clinical trials lists recorded in the patient population

| NCT # (Phase) | Trial Name | Population | Intervention | Primary Endpoint |
| --- | --- | --- | --- | --- |
| CTR20201637  (II) | A clinical study evaluating the safety and preliminary efficacy of FCN-437c in the treatment of women with ER-positive, HER2-negative advanced breast cancer | HR+, HER2-advanced breast cancer patients | FCN-437c | ORR |
| IMpassion131  (III) | A Study of Atezolizumab and Paclitaxel Versus Placebo and Paclitaxel in Participants with Previously Untreated Locally Advanced or Metastatic Triple Negative Breast Cancer (TNBC) | Previously untreated inoperable locally advanced or metastatic triple negative breast cancer | Atezolizumab, Paclitaxel | PFS |
| PAUSE(III) | A Study of Pertuzumab in Combination with Trastuzumab (Herceptin) and a Taxane in First-Line Treatment in Participants with Human Epidermal Growth Factor 2 (HER2)-Positive Advanced Breast Cancer (PERUSE) | (HER2)-Positive advanced breast cancer | Docetaxel, Nab-paclitaxel,  Paclitaxel,  Pertuzumab,Trastuzumab | TEAEs |
| GENORGB221-003(III) | Clinical Study of Recombinant Anti-HER2 Humanized Monoclonal Antibody (GB221) for Injection | HER-2-positive advanced breast cancer | GB221, Capecitabine | PFS |
| D6997L00021  (III) | Comparing the Efficacy and Tolerability of Fulvestrant 500 mg Versus 250 mg in Advanced Breast Cancer Women | advanced breast cancer women | Fulvestrant | PFS |
| BELLE-2(III) | Phase III Study of BKM120/Placebo with Fulvestrant in Postmenopausal Patients with Hormone Receptor Positive HER2-negative Locally Advanced or Metastatic Breast Cancer Refractory to Aromatase Inhibitor (BELLE-2) | HR+, HER2 - locally advanced or metastatic breast cancer | Fulvestrant BKM120 | PFS |
| DESTINY-Breast09(III) | Trastuzumab Deruxtecan (T-DXd) With or Without Pertuzumab Versus Taxane, Trastuzumab and Pertuzumab in HER2-positive Metastatic Breast Cancer (DESTINY-Breast09) | HER2-positive metastatic breast cancer | Trastuzumab deruxtecan, Pertuzumab, Taxane,Trastuzumab | PFS |
| NCT03863223  (III) | A Study of Pyrotinib in Combination with Trastuzumab and Docetaxel in Patients with HER2 Metastatic Breast Cancer | HER2+ metastatic breast cancer | pyrotinib, trastuzumab, docetaxel | PFS |
| NCT01920568  (III) | A Study to Compare Denosumab With Zoledronic Acid in Subjects with Bone Metastases from Solid Tumors | subjects with bone metastases from solid tumors | Denosumab | uNTx/uCr* |

***Abbreviation:*** *ORR: objective response rate, TEAEs: Treatment-emergent adverse events, PFS: Progression-free survival*

**uNTx/uCr: Percent Change (Chg) From Baseline (BL) to Week (Wk)13 in Urinary Amino-terminal Cross-linking Telopeptide of Type I Collagen Corrected for Urine Creatinine (uNTx/uCr)*

Supplementary Table 2. Clinicopathological and treatment characteristics associated with receiving courses of intracranial RT in BCBM patients

| Characteristics | **number of courses of intracranial RT** | | | ***p* value** |
| --- | --- | --- | --- | --- |
|  | **0**  **n(**%**)** | **1**  **n(**%**)** | **≥2**  **n(**%**)** |  |
| No MDT | 16(7.6) | 84(39.8) | 6(2.8) | ＜0.001 |
| MDT | 10(4.7) | 55(26.0) | 40(18.9) | |
| Molecular subtype |  |  |  | < 0.001 |
| A | 2(1) | 20(9.5) | 1(0.5) |  |
| B(HER2−) | 8(3.8) | 26(12.3) | 11(5.2) |  |
| B(HER2+) | 5(2.4) | 23(10.9) | 11(5.2) |  |
| HER2+ | 5(2.4) | 33(15.6) | 9(4.3) |  |
| TN | 6(2.8) | 37(17.5) | 14(6.6) |  |
| Clinical stage |  |  |  | 0.229 |
| I | 1(0.5) | 16(7.6) | 4(1.9) |  |
| II | 7(3.3) | 50(23.7) | 18(15) |  |
| III | 9(4.3) | 54(25.6) | 15(7.1) |  |
| IV | 9(4.3) | 19(9) | 9(4.3) |  |
| Pathological type |  |  |  | 0.439 |
| IDC | 26(12.3) | 132(62.6) | 43(20.4) |  |
| no IDC | 0 | 7(3.3) | 3(1.4) |  |
| Histological grade |  |  |  | 0.314 |
| I~II | 12(5.7) | 85(40.3) | 29(13.7) |  |
| III | 14(6.6) | 54(25.6) | 17(8.1) |  |
| Primary surgery |  |  |  | < 0.001 |
| BCS | 4(1.9) | 7(3.3) | 10(4.7) |  |
| MRM | 13(6.2) | 113(53.6) | 27(12.8) |  |
| Only biopsy | 9(4.3) | 19(9) | 9(4.3) |  |
| NAST /and AST |  |  |  |  |
| Unknown | 2(0.9) | 1(0.5) | 1(0.5) | 0.175 |
| No | 6(2.8) | 31(14.7) | 8(3.8) |  |
| Yes | 18(8.5) | 107(50.7) | 37(17.5) |  |
| Age(y) |  |  |  | 0.88 |
| ＜60 | 18(8.5) | 90(52.7) | 31(14.7) |  |
| ≥60 | 8(3.8) | 49(23.2) | 15(7.1) |  |
| Infratentorial |  |  |  | 0.205 |
| No | 10(4.7) | 49(23.2) | 23(10.9) |  |
| Yes | 16(7.6) | 90(42.7) | 23(10.9) |  |
| Number of BM lesions | |  |  | 0.627 |
| 1 | 5(2.4) | 33(15.6) | 8(3.8) |  |
| ＞1 | 21(10) | 106(50.2) | 38(18) |  |
| Clinical symptom |  |  |  | 0.35 |
| Absence | 6(2.8) | 26(12.3) | 5(2.4) |  |
| Presence | 20(9.5) | 113(53.6) | 41(19.4) |  |
| modified Breast-GPA score | |  |  | 0.257 |
| meningeal metastases & 0-1 | 12(6.2) | 57(27) | 12(6.2) |  |
| 1.5-2 | 6(2.8) | 52(24.6) | 22(10.4) |  |
| ≥2.5 | 7(3.3) | 30(14.2) | 11(5.2) |  |
| Extracranial metastases | |  |  | 0.546 |
| No | 6(2.8) | 23(10.9) | 6(2.8) |  |
| Yes | 20(9.5) | 116(55) | 40(19) |  |
| Intracranial RT at initial BM | |  |  | ＜0.001 |
| No RT | 26(12.3) | 0 | 0 |  |
| WBRT only | 0 | 105(49.8) | 16(7.6) |  |
| SRS/WBRT-HA | 0 | 34(16.1) | 30(14.2) |  |
| Prospective clinical trial |  |  |  | 0.023 |
| No | 19(9) | 120(56.9) | 32(15.2) |  |
| Yes | 7(3.3) | 19(9) | 14(6.6) |  |
| New drugs |  |  |  | 0.069 |
| No | 20(9.5) | 111(52.6) | 29(13.7) |  |
| Yes | 6(2.8) | 28(13.3) | 17(8.1) |  |
| Re-biopsy |  |  |  | 0.048 |
| No | 18(8.5) | 67(31.8) | 18(8.5) |  |
| Yes | 8(3.8) | 72(34.1) | 28(13.3) |  |

***Abbreviations:*** *RT: radiotherapy; MDT: multidisciplinary team; A: Luminal A; B(HER2−): Luminal B (HER2−); B(HER2+): Luminal B (HER2+); HER‐2+: HER‐2 overexpression; TN: Triple‐negative; ER/PR: Estrogen or progesterone receptor; IDC: invasive ductal carcinoma; BCS: Breast conserving therapy; MRM: Modified radical mastectomy; NAST: Neo‐adjuvant systemic therapy; AST: adjuvant systemic therapy; BM: brain metastasis; KPS: Karnofsky performance status; mBreast-GPA score: modified Graded Prognostic Assessment score; WBRT: whole brain radiation therapy; WBRT-HA: WBRT with hippocampal avoidance; SRS: stereotactic radiosurgery; fSRT: fractionated stereotactic radiotherapy*

Supplementary Table 3. Clinicopathological and treatment characteristics associated with radiotherapeutic techniques

| Characteristics | | Radiotherapeutic Techniques | | | *p* value |
| --- | --- | --- | --- | --- | --- |
|  | **No RT**  **n(**%**)** | | **WBRT only**  **n(**%**)** | **SRS/WBRT-HA**  **n(**%**)** |  |
| No MDT | | 16(7.6) | 72(34.1) | 18(8.5) | *< 0.001* |
| MDT | | 10(4.7) | 34(16.1) | 61(28.9) |  |
| Molecular subtype | | |  |  | 0.126 |
| A | | 2(1) | 18(8.5) | 3(1.4) |  |
| B(HER2−) | | 8(3.8) | 18(8.5) | 19(9) |  |
| B(HER2+) | | 5(2.4) | 16(7.6) | 18(8.5) |  |
| HER2+ | | 5(2.4) | 22(10.4) | 20(9.5) |  |
| TN | | 6(2.8) | 32(15.2) | 19(9) |  |
| Clinical stage | |  |  |  | 0.172 |
| I | | 1(0.5) | 14(6.6) | 6(2.8) |  |
| II | | 7(3.3) | 37(17.5) | 31(14.7) |  |
| III | | 9(4.3) | 41(19.4) | 28(13.3) |  |
| IV | | 9(4.3) | 14(6.6) | 14(6.6) |  |
| Pathological type | | |  |  | 0.47 |
| IDC | | 26(12.3) | 100(47.4) | 75(35.5) |  |
| no IDC | | 0 | 6(2.8) | 4(1.9) |  |
| Histological grade | | |  |  | 0.297 |
| I~II | | 12(5.7) | 64(30.3) | 50(23.7) |  |
| III | | 14(6.6) | 42(19.9) | 29(13.7) |  |
| Primary surgery | | |  |  | 0.01 |
| BCS | | 4(1.9) | 6(2.8) | 11(5.2) |  |
| MRM | | 13(6.2) | 86(40.8) | 54(25.6) |  |
| Only biopsy | | 9(4.3) | 14(6.6) | 14(6.6) |  |
| NAST /and AST | | |  |  | 0.207 |
| Unknown | | 2(0.9) | 1(0.5) | 1(0.5) |  |
| No | | 6(2.8) | 24(11.4) | 15(7.1) |  |
| Yes | | 18(8.5) | 81(38.4) | 63(29.9) |  |
| Age(y) | |  |  |  | 0.71 |
| ＜60 | | 18(8.5) | 67(31.8) | 54(25.6) |  |
| ≥60 | | 8(3.8) | 39(18.5) | 25(11.8) |  |
| Infratentorial | |  |  |  | 0.042 |
| No | | 10(4.7) | 33(15.6) | 39(18.5) |  |
| Yes | | 16(7.6) | 73(34.6) | 40(19) |  |
| Number of BM lesions | | |  |  | 0.885 |
| 1 | | 5(2.4) | 23(10.9) | 20(9.5) |  |
| 2-4 | | 12(5.7) | 54(25.6) | 40(19) |  |
| ＞4 | | 9(4.3) | 29(13.7) | 19(9) |  |
| Clinical symptom | | |  |  | 0.73 |
| Absence | | 6(2.8) | 18(8.5) | 13(6.2) |  |
| Presence | | 20(9.5) | 88(47.1) | 66(31.3) |  |
| modified Breast-GPA score | | |  |  | 0.072 |
| meningeal metastases & 0-1 | | 12(6.2) | 48(22.7) | 22(10.4) |  |
| 1.5-2 | | 6(2.8) | 37(17.5) | 37(17.5) |  |
| ≥2.5 | | 7(3.3) | 21(10) | 20(9.5) |  |
| Extracranial metastases | | |  |  | 0.547 |
| No | | 6(2.8) | 18(8.5) | 11(5.2) |  |
| Yes | | 20(9.5) | 88(41.7) | 68(32.2) |  |
| Prospective clinical trial | | |  |  | 0.018 |
| No | | 19(9) | 94(44.5) | 58(27.5) |  |
| Yes | | 7(3.3) | 12(5.7) | 21(10) |  |
| New drugs | |  |  |  | < 0.001 |
| No | | 20(9.5) | 92(43.6) | 48(22.7) |  |
| Yes | | 6(2.8) | 14(6.6) | 31(14.7) |  |
| Re-biopsy | |  |  |  | 0.051 |
| No | | 18(8.5) | 46(21.8) | 39(18.5) |  |
| Yes | | 8(3.8) | 60(28.4) | 40(19) |  |

***Abbreviations:*** *RT: radiotherapy; MDT: multidisciplinary team; A: Luminal A; B(HER2−): Luminal B (HER2−); B(HER2+): Luminal B (HER2+); HER‐2+: HER‐2 overexpression; TN: Triple‐negative; BCS: Breast conserving therapy; MRM: Modified radical mastectomy; BM: brain metastasis; WBRT: whole brain radiation therapy; WBRT-HA: WBRT with hippocampal avoidance; SRS: stereotactic radiosurgery; fSRT: fractionated stereotactic radiotherapy*

Supplementary Table 4. Clinicopathological and treatment characteristics associated with prospective clinical trials

| Characteristics | **no PCT**  **n(**%**)** | **PCT**  **n(**%**)** | *p* value |
| --- | --- | --- | --- |
| No MDT | 98(46.4) | 8(3.8) | < 0.001 |
| MDT | 73(34.6) | 32(15.2) |  |
| Molecular subtype | |  | 0.05 |
| A | 20(9.5) | 3(1.4) |  |
| B(HER2−) | 34(16.1) | 11(5.2) |  |
| B(HER2+) | 26(12.3) | 13(6.2) |  |
| HER2+ | 41(19.4) | 6(2.8) |  |
| TN | 50(23.7) | 7(3.3) |  |
| ER/PR |  |  | 0.8 |
| Negative | 85(40.3) | 19(9) |  |
| Positive | 86(40.8) | 21(10) |  |
| HER2 status | |  | 0.017 |
| Negative | 108(51.2) | 17(8.1) |  |
| Positive | 63(29.9) | 23(10.9) |  |
| Clinical stage | |  | 0.172 |
| I | 15(7.1) | 6(2.8) |  |
| II | 59(28) | 16(7.6) |  |
| III | 69(32.7) | 9(4.3) |  |
| IV | 28(13.3) | 9(4.3) |  |
| Pathological type | |  | 0.93 |
| IDC | 163(77.3) | 38(18) |  |
| no IDC | 8(3.8) | 2(0.9) |  |
| Histological grade | |  | 0.029 |
| I~II | 96(45.5) | 30(14.2) |  |
| III | 75(35.5) | 10(4.7) |  |
| Primary surgery | |  | 0.60 |
| BCS | 18(8.5) | 3(1.4) |  |
| MRM | 125(59.2) | 28(13.3) |  |
| Only biopsy | 28(13.3) | 9(4.3) |  |
| NAST /and AST | |  | 0.014 |
| Unknown | 1(0.5) | 3(1.4) |  |
| No | 38(18) | 7(3.3) |  |
| Yes | 132(62.6) | 30(14.2) |  |
| Age(y) |  |  | 0.81 |
| ＜60 | 112(53.1) | 27(12.8) |  |
| ≥60 | 59(28) | 13(6.2) |  |
| Infratentorial | |  | 0.376 |
| No | 64(30.3) | 18(8.5) |  |
| Yes | 107(50.7) | 22(10.4) |  |
| Number of BM lesions | |  | 0.386 |
| 1 | 42(19.9) | 6(2.8) |  |
| 2-4 | 85(40.3) | 21(10) |  |
| ＞4 | 44(20.9) | 13(6.2) |  |
| Clinical symptom | |  | 0.043 |
| Absence | 22(10.4) | 1(0.5) |  |
| Presence | 125(59.2) | 39(18.5) |  |
| modified Breast-GPA score | | | 0.864 |
| meningeal metastases & 0-1 | 66(31.3) | 17(8.1) |  |
| 1.5-2 | 65(30.8) | 15(7.1) |  |
| ≥2.5 | 40(19) | 8(3.8) |  |
| extracranial metastases | |  | 0.519 |
| No | 27(12.8) | 8(3.8) |  |
| Yes | 144(68.2) | 32(15.2) |  |
| Re-biopsy | |  | 0.054 |
| No | 78(37) | 25(11.8) |  |
| Yes | 93(44.1) | 15(7.1) |  |
| Posterior systemic therapy lines after initial BM | | | 0.025 |
| 0 | 47(22.3) | 3(1.4) |  |
| 1 | 62(29.4) | 17(8.1) |  |
| ≥2 | 62(29.4) | 20(9.5) |  |

***Abbreviations:*** *RT: radiotherapy; MDT: multidisciplinary team; A: Luminal A; B(HER2−): Luminal B (HER2−); B(HER2+): Luminal B (HER2+); HER‐2+: HER‐2 overexpression; TN: Triple‐negative; ER/PR: Estrogen or progesterone receptor; IDC: invasive ductal carcinoma; BCS: Breast conserving therapy; MRM: Modified radical mastectomy; NAST: Neo‐adjuvant systemic therapy; AST: adjuvant systemic therapy; BM: brain metastasis; KPS: Karnofsky performance status; mBreast-GPA score: modified Graded Prognostic Assessment score; WBRT: whole brain radiation therapy; WBRT-HA: WBRT with hippocampal avoidance; SRS: stereotactic radiosurgery; fSRT: fractionated stereotactic radiotherapy*
